# The prediction of student wellbeing and flourishing from university learning experiences

**DOI:** 10.3389/fpsyg.2025.1585058

**Published:** 2025-07-24

**Authors:** Yue Zhao, Yunqing Hua

**Affiliations:** Teaching and Learning Innovation Centre, The University of Hong Kong, Pokfulam, Hong Kong SAR, China

**Keywords:** student wellbeing, flourishing, learning experience, hedonic wellbeing, eudaimonic wellbeing, social wellbeing

## Abstract

Student wellbeing and flourishing are becoming increasingly essential in the evolving landscape of higher education. The current study explores the relationship between university students’ learning experiences and their hedonic, eudaimonic, and social wellbeing. Employing structural equation modelling and regression analyses, the study involves a total of 5,505 undergraduate students from a Hong Kong university, spanning junior to senior years and a wide range of academic disciplines. Findings reveal that students’ hedonic, eudaimonic, and social wellbeing are significantly predicted by their learning experiences. Notably, internal factors of students’ learning experiences including cognitive, social, and value developments demonstrate a stronger prediction, while external factors of students’ learning experiences like clear goals and standards, good teaching, feedback, and assessment also significantly contribute to students’ wellbeing. The study extends the current literature and offer new insights into the wellbeing benefits of student learning experiences and underscores the need for holistic strategies and tailored approaches to nurture student flourishing in higher education.

## 1 Introduction

In the evolving landscape of higher education, there is an increasing emphasis on the multifaceted objectives that universities aim to achieve for students. Traditionally, the primary aim of university education has been to develop students’ knowledge, understanding, and cognitive capacities, preparing them for employment. However, there is a growing recognition that education’s role in promoting student wellbeing and flourishing extends beyond cognitive development (Oades et al., 2011; Ellyatt, 2022; Vander Weele, 2022; Kristjánsson, 2023; Curren et al., 2024; Kern et al., 2024). Flourishing, closely related to wellbeing, involves achieving an optimal state where various aspects of a student’s life are thriving (de Ruyter et al., 2024). In higher education, flourishing implies that students are not only surviving but thriving; they are intellectually stimulated, emotionally resilient, and socially connected. In recent years, it has been argued that flourishing should be viewed not only as a significant aim but even as the central aim of all educational endeavors (Kristjánsson and Vander Weele, 2024). In addition to academic achievements, universities should prioritize the holistic development of students, encompassing their social, emotional, and character-related capacities (Vander Weele and Hinton, 2024). Such an emphasis on student wellbeing and flourishing holds the promise of better preparing students not only for academic success but also for leading fulfilling lives and contributing meaningfully to society, underscoring that university is not just a pathway to better jobs; it is also a means to better lives (OECD, 2012). Achieving a high state of wellbeing, or student flourishing, is crucial for students to thrive both academically and personally.

Universities have been increasingly implementing initiatives that address and assess various aspects of student wellbeing, recognizing its critical role in fostering academic and personal flourishing. These initiatives include positive psychology interventions (e.g., Hall et al., 2024), mental health services (e.g., Osborn et al., 2022), curriculum-embedded activities (e.g., Upsher et al., 2022), and wellbeing assessments (e.g., Williams et al., 2017). Universities profoundly influence students’ learning experiences, making it crucial to comprehensively understand how university experience, as one of the key factors, shapes student wellbeing and flourishing in order to inform effective institutional practices and policies (Baik et al., 2019; Campbell et al., 2022; Gilmore et al., 2025; Riva et al., 2020).

### 1.1 University students’ wellbeing

Defining wellbeing is complex and multifaceted, with multiple theoretical frameworks present in the literature (Marsh et al., 2020). While definitions of wellbeing vary across frameworks and in the higher education context (e.g., Barkham et al., 2019; Dodd et al., 2021), these frameworks often distinguish between hedonic and eudaimonic wellbeing. Hedonic wellbeing primarily involves happiness and life satisfaction, focusing on the cognitive and affective facets of wellbeing, such as subjective experiences of joy, contentment, and overall life satisfaction, as posited by Diener’s (1984) theory of subjective wellbeing. Eudaimonic wellbeing, rooted in the concept of living a fulfilling and meaningful life (Steger, 2009), involves realizing one’s potential and pursuing personal growth (Ryff, 1989; Ryan and Deci, 2001). Additionally, social wellbeing is another crucial dimension, particularly in a university context (Eloff et al., 2022; Makaremi et al., 2024; Lemyre et al., 2024; Liu et al., 2021; Stallman et al., 2018). Social wellbeing entails social connections and a sense of belonging within a community. Altogether, hedonic, eudaimonic, and social wellbeing provide a multifaceted framework for understanding students flourishing, emphasizing the importance of feeling good, functioning well, and being socially connected. Psychological indicators such as positive emotions and life satisfaction exemplify hedonic wellbeing; a sense of meaning, purposeful goal orientation, and self-realization characterize eudaimonic wellbeing; while feelings of social connectedness, community, and belonging underpin social wellbeing (Henderson et al., 2013; Joshanloo, 2016). Growing evidence suggests that students with higher levels of wellbeing tend to achieve better academic outcomes, maintain healthier lifestyles, and demonstrate increased resilience (Durlak et al., 2011; Lyubomirsky et al., 2005; Tejada-Gallardo et al., 2020). Student wellbeing has also been linked to their engagement and performance in curricular, co-curricular, and extracurricular activities in universities (Cox and Brewster, 2020; Plakhotnik et al., 2021). Recent work highlights the link between learning and wellbeing across contexts, from school-based studies (Collie and Hascher, 2024) to holistic models like art-of-living (Schmitz et al., 2022).

To explain the linkage between learning and wellbeing, several psychological theories offer conceptual grounding. Self-Determination Theory (Ryan and Deci, 2000) posits that wellbeing is enhanced when students experience autonomy, competence, and relatedness—core psychological needs that can be cultivated through meaningful learning experiences. The broaden-and-build theory (Fredrickson, 2001) explains how positive emotions, often triggered by meaningful learning, can expand cognitive and behavioral repertoires and build psychological resources. In addition, theories of social wellbeing (e.g., Keyes, 1998) emphasize the importance of social integration, acceptance, and coherence as central components of wellbeing. Together, these theories underscore the multifaceted pathways through which learning experiences contribute to student wellbeing and flourishing. Complementing these frameworks, educational models such as Biggs’ 3P model (Biggs and Tang, 2011; Kember et al., 2020) and Behavioral Learning Theory (de la Fuente-Arias, 2017) emphasize that quality teaching and supportive environments play a critical role in students’ cognitive, social, and value developments. Together, these perspectives illuminate the positive associations between student wellbeing and learning experiences.

### 1.2 University students’ learning experiences and wellbeing

The conceptualization of the university student experience can be traced back to two primary lenses (Fryer et al., 2021; Zeng et al., 2023). The first lens, originating in Great Britain, focused on the relationship between learners’ cognitive processing and their perceptions of assessment. This led to the development of large-scale surveys like the Course Experience Questionnaire, which assesses students’ cognitive processing and perceptions of learning environments and has been widely used in Australia, the United Kingdom, and Hong Kong (Entwistle and Ramsden, 1983; Ramsden, 1991; Lizzio et al., 2002; Sun and Richardson, 2012; Webster et al., 2009; Zhao et al., 2017a,b). The second lens, prevalent in the United States, gave rise to the development of a theory of student engagement and the National Survey of Student Engagement (Kuh et al., 1997). According to Kuh (2009), university engagement involves the amount of time and effort students invest in activities that are empirically linked to desirable university educational outcomes, as well as the strategies universities use to encourage student engagement in these activities.

Despite their differences, these two traditions share the ultimate goal of understanding and enhancing the student learning experiences. The learning experiences of university students involve the synergy of internal and external factors that shape students’ university experience and engagement. Internally, it involves students’ cognitive, social, and value development as they engage in academic and extracurricular activities both inside and outside the classroom. Externally, it includes the learning environment, such as students’ perceptions of assessment, quality teaching, feedback, and clear goals and standards provided by teachers to foster student engagement and achieve desirable educational outcomes. Both internal and external factors of the student learning experiences play a substantial role in wellbeing (Baik et al., 2019; Gilmore et al., 2025).

Research highlights the importance of internal factors in student wellbeing. Academic competencies such as critical thinking, problem-solving, and teamwork have been linked to positive outcomes across multiple wellbeing domains, including physical, social, and environmental wellbeing (Baumann et al., 2014; Vázquez-Parra et al., 2023). Academic and social experiences also play a central role in fostering wellbeing (Brooker and Vu, 2020), particularly through students’ sense of purpose and meaningful engagement in learning (Stanton et al., 2016). Social participation and a sense of community further support wellbeing across diverse university contexts (Cicognani et al., 2008). In Chinese higher education, internal aspects of engagement—such as academic challenge and learning with peers—have been shown to mediate the link between personal wellbeing and academic performance (Yu et al., 2018).

External factors also matter to student wellbeing. A growing body of qualitative research emphasizes the importance of good teaching, pedagogy, and assessment practices—including timely and effective feedback—as key to supporting wellbeing (Upsher et al., 2023; Baik et al., 2019). Feedback, in particular, plays a dual role: while it supports learning cognitively, it can also evoke emotional responses that affect wellbeing, highlighting its complex impact (Jones et al., 2020; Turnbull, 2022). Studies across the UK, Canada, and African higher education systems consistently show that supportive teaching environments, strong teacher-student relationships, and accessible student services contribute to student wellbeing (Riva et al., 2020; Lindsay et al., 2023; Eloff et al., 2022). In Chinese universities, where peer and teacher support are crucial, a supportive environment can further influence student wellbeing (Zou et al., 2024).

### 1.3 The current study

Overall, the literature underscores the multifaceted nature of student wellbeing, emphasizing the complexity and dynamics of internal and external factors of student learning experiences in shaping student wellbeing and flourishing. While valuable insights have been gained from existing studies—many of which as reviewed earlier are based on qualitative methods—their findings can be difficult to generalize statistically supported conclusions. Additionally, many studies have examined wellbeing as a global measure, leaving a gap in understanding how learning experience factors affect specific dimensions of wellbeing. To address these research gaps, the current study is designed to investigate the following research questions: to what extent do university students’ learning experiences predict their hedonic, eudaimonic, and social wellbeing? We hypothesize that both internal and external learning experiences will positively predict all three dimensions of wellbeing.

The significance of the study is manifold. It extends the current literature on students’ wellbeing and learning experiences by providing new empirical evidence and useful insights into the wellbeing benefits of student learning. From a pedagogical perspective, this study sheds light on effective academic practices, teaching methods, and learning environments that can enhance student wellbeing. Psychologically, it provides a deeper understanding of how various dimensions of wellbeing are influenced by learning experiences. Practically, the findings will guide higher education institutions in developing targeted policies and interventions to enhance student wellbeing and learning. Overall, this study aims to contribute to the development of holistic strategies to nurture student flourishing in higher education.

## 2 Methods

### 2.1 Participants

All undergraduate students in their first, second, and final years at a Hong Kong university were invited to participate in the study. The final sample included 5,505 students, with a representative gender ratio of 58% female. Participants ranged in age from 18 to 24, spanning junior to senior years. This sample represents approximately 47% of the undergraduate student population and encompasses all academic faculties and disciplines within the university.

### 2.2 Measures

The Student Learning Experience Questionnaire (SLEQ; Zhao et al., 2017a,b) was employed to assess students’ wellbeing and learning experiences. The SLEQ includes scales that measure hedonic, eudaimonic, and social wellbeing, as well as students’ perceptions of their learning experiences, covering both internal and external factors. Respondents rated their agreement with each SLEQ item on a five-point Likert scale (1 = strongly disagree to 5 = strongly agree), with higher ratings indicating more positive perceptions. The SLEQ has demonstrated adequate reliability and validity (Zhao et al., 2017a,b).

Students’ wellbeing was assessed by nine items capturing hedonic, eudaimonic, and social wellbeing. Sample questions included “I feel happy” (hedonic wellbeing), “My life is valuable and worthwhile” (eudaimonic wellbeing), and “I feel I am really part of the university” (social wellbeing). In the current study, the wellbeing scales exhibited Cronbach’s alpha values ranging from 0.86 to 0.94. The internal factors of university learning experiences were assessed by 19 items related to students’ perceptions of their attainment of educational outcomes, which fell into three domains: cognitive (knowledge pursuit, critical thinking, lifelong learning, and problem-solving; Cronbach’s alpha of 0.93), social (empathetic understanding, intercultural skills, communication skills, and collaboration; Cronbach’s alpha of 0.91), and value (personal integrity and ethics, global perspective, and civic commitment; Cronbach’s alpha of 0.88). The external factors of university learning experiences were assessed by 12 items related to students’ perceptions of the learning environment, which fell into four domains: clear goals and standards (Cronbach’s alpha of 0.90), good teaching (Cronbach’s alpha of 0.86), feedback from teachers (Cronbach’s alpha of 0.93), and assessment for understanding (Cronbach’s alpha of 0.91).

### 2.3 Analysis procedure

Data were collected via an online survey administered near the end of the academic year. Students were invited through university emails and the university’s student portal. Informed consent was obtained before participation. Ethical approval was granted by the university’s Human Research Ethics Committee.

To gain an overall picture of the associations between students’ wellbeing and their university learning experiences, structural equation modeling (SEM) was applied, where students’ wellbeing, internal factors of learning experiences, and external factors of learning experiences were modelled as three latent variables. The latent variable of wellbeing was specified using three observed indicators—hedonic, eudaimonic, and social wellbeing—to allow a parsimonious model that reflects the interconnected nature of these components while accounting for measurement error. The latent internal factors variable was formed from students’ average scores on cognitive, social, and value development domains; the latent external factors variable was based on average scores across clear goals and standards, good teaching, feedback from teachers, and assessment for understanding. All observed variables demonstrated adequate reliability (Cronbach’s alpha > 0.80) and were supported by factor analytic evidence. The SEM analysis was conducted using Mplus 8 (Muthén and Muthén, 2017). Based on the literature (Bentler and Bonett, 1980; Browne and Cudeck, 1993; Hu and Bentler, 1999; Lance et al., 2006), a good model fit is typically indicated by a comparative fit index (CFI) greater than 0.90, a Tucker-Lewis index (TLI) greater than 0.90, and a Root Mean Square Error of Approximation (RMSEA) less than 0.06, and a Standardized Root Mean Square Residual (SRMR) less than 0.08.

Further, a multivariate linear regression model was utilized to yield a more detailed examination of the associations. Specifically, this analysis quantified each internal and external factor of university learning experiences in predicting the hedonic, eudaimonic, and social dimensions of students’ wellbeing, while controlling for demographic variables including gender, academic discipline (e.g., arts, science, medicine, business and law), student year (first, second, and final years), and student status (local and non-local students). To check for multicollinearity, bivariate correlations and variance inflation factors (VIF) were examined. All VIF values were below 3, indicating no serious multicollinearity concerns.

## 3 Results

**Figure 1:**
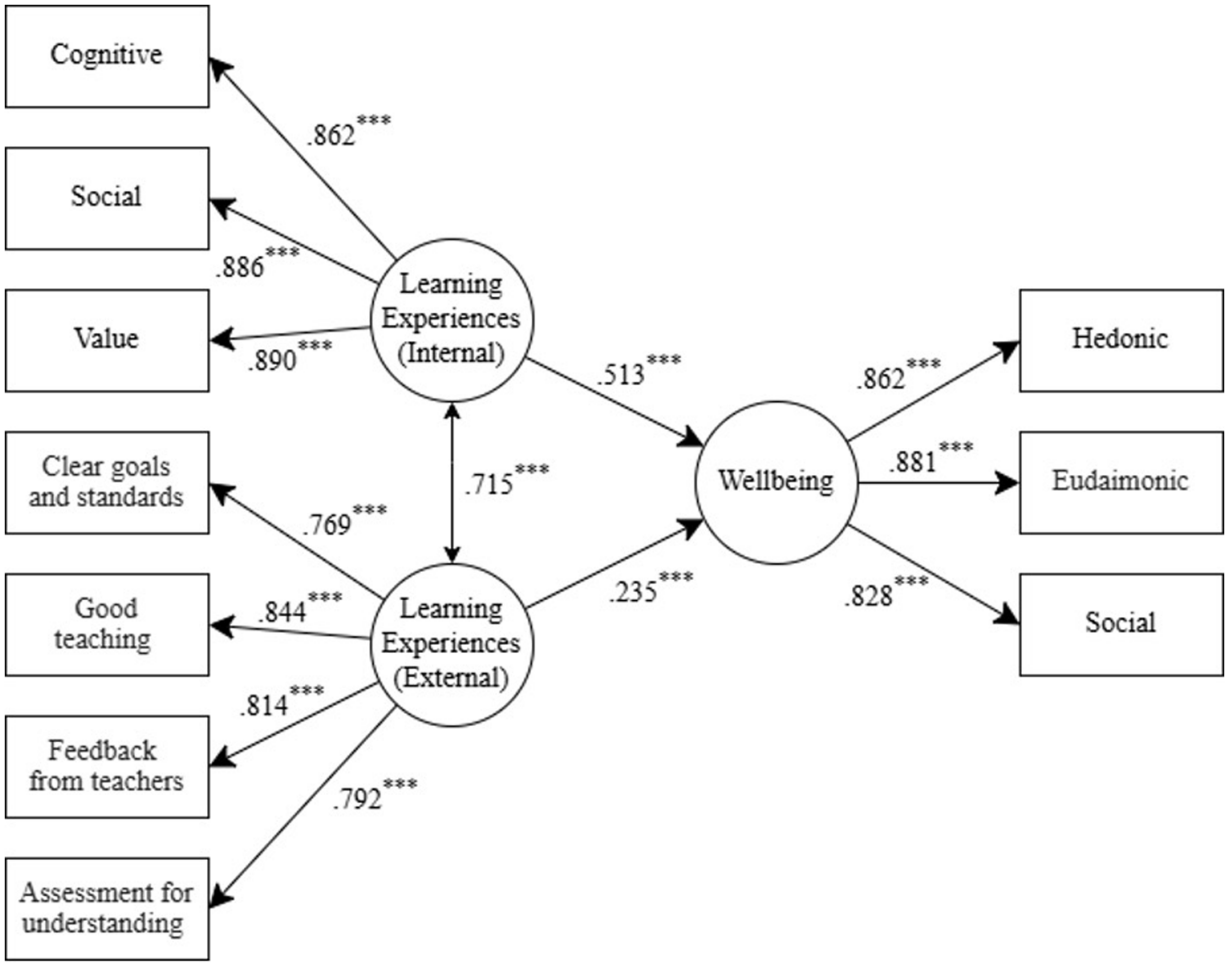
Students’ wellbeing and university learning experiences through structural equation modeling (SEM). All parameter estimates from the SEM are reported based on standardized solutions. ^***^ *p* < 0.001.

From the SEM (Figure 1), students’ wellbeing was significantly associated with internal factors (*β* = 0.51, *p* < 0.001) and external factors (*β* = 0.24, *p* < 0.001) of university learning experiences (CFI = 0.96, TLI = 0.94, RMSEA = 0.074, SRMR = 0.038), with internal factors showing a stronger association. From the regression analysis (Table 1), focusing on hedonic wellbeing, internal factors containing students’ cognitive and value developments, and external factors including clear goals and standards, feedback from teachers, and good teaching were significant predictors of students’ hedonic wellbeing. Regarding eudaimonic wellbeing, internal factors including students’ cognitive, social, and value developments, and external factors like clear goals and standards, good teaching, and assessment for understanding were significant predictors of students’ eudaimonic wellbeing. For social wellbeing, internal factors including students’ cognitive, social, and value developments, and external factors such as clear goals and standards and feedback from teachers were significant predictors of students’ social wellbeing. Overall, various internal and external factors of university learning experiences significantly predicted different dimensions of students’ wellbeing, with internal factors generally showing stronger predictions.

**Table 1:** Multivariate linear regression analysis of university learning experiences as predictors of students’ wellbeing.

Predictors	Wellbeing: hedonic			Wellbeing: eudaimonic			Wellbeing: social		
	β	SE	*p*	β	SE	*p*	β	SE	*p*
Students’ learning experiences: internal factors									
Cognitive	0.254^***^	0.026	<0.001	0.251^***^	0.025	<0.001	0.139^***^	0.026	<0.001
Social	−0.004	0.029	0.879	0.093^**^	0.028	0.001	0.157^***^	0.029	<0.001
Value	0.152^***^	0.027	<0.001	0.182^***^	0.028	<0.001	0.209^***^	0.028	<0.001
Students’ learning experiences: external factors									
Clear goals and standards	0.135^***^	0.021	<0.001	0.059^**^	0.020	0.003	0.076^***^	0.021	<0.001
Good teaching	0.065^**^	0.023	0.004	0.061^**^	0.021	0.004	0.035	0.022	0.105
Feedback from teacher	0.067^**^	0.021	0.002	0.011	0.021	0.599	0.087^***^	0.021	<0.001
Assessment for understanding	0.018	0.022	0.412	0.062^**^	0.021	0.003	0.020	0.022	0.356

All regression coefficients (β) are reported based on standardized solutions. ^**^ *p* < 0.01, ^***^ *p* < 0.001.

## 4 Discussion

The current study provides empirical evidence showing that students’ wellbeing is significantly predicted by both internal and external factors of university learning experiences, with internal factors showing a stronger prediction. Internal factors including cognitive, social, and value developments, and external factors like clear goals and standards, good teaching, and feedback from teachers, were significant predictors of hedonic, eudaimonic, and social wellbeing. These findings emphasize the importance of a tailored approach to examining various dimensions of students’ wellbeing within a university learning environment.

The present findings offer empirical support for several foundational psychological and educational theories that explain the connection between learning and wellbeing. The strong predictive role of internal factors supports Self-Determination Theory, which posits that autonomy, competence, and relatedness are critical for wellbeing (Ryan and Deci, 2000). The contribution of external factors echoes educational models such as Biggs’ 3P model (Biggs and Tang, 2011; Kember et al., 2020), emphasizing the role of the learning environment. By identifying which aspects of learning experiences are most closely tied to different wellbeing dimensions, this study offers nuanced support for and adds empirical depth to these theoretical frameworks. By identifying which aspects of learning experiences are most strongly associated with distinct dimensions of wellbeing, this study advances existing theoretical frameworks by adding empirical depth and providing new insights into how university learning contributes not only to academic outcomes but also to students’ wellbeing and flourishing.

### 4.1 Students’ hedonic, eudaimonic, and social wellbeing

Findings of the study show that, among the three dimensions of wellbeing, students’ learning experiences most strongly predicted eudaimonic wellbeing, followed by hedonic and social wellbeing. This finding aligns with the growing recognition that eudaimonia—encompassing constructs such as personal growth and meaning—reflects a deeper form of psychological functioning and flourishing than the more experience-based nature of hedonia (Huta and Waterman, 2014). As such, eudaimonic wellbeing is likely to be more directly influenced by meaningful learning experiences that cultivate students’ values, competencies, and holistic development.

From a Self-Determination Theory perspective (Ryan and Deci, 2000), eudaimonic wellbeing is fostered when students’ needs for autonomy, competence, and relatedness are met. Internal learning experiences—such as cognitive development (e.g., critical thinking), social development (e.g., communication and collaboration), and value development (e.g., integrity and civic commitment)—correspond to these needs and support students’ self-directed growth. External factors such as assessments for understanding, good teaching, and clear goals and standards further enhance the learning environment by reinforcing competence, autonomy, and connection. These internal and external factors collectively contribute to enhanced eudaimonic wellbeing by providing intrinsic drivers and external supports that catalyze student flourishing.

The influences of students’ learning experiences on hedonic and social wellbeing are also significant. Hedonic wellbeing, encompassing pleasure, happiness, and life satisfaction, is predicted by both internal and external factors within the university environment. According to the broaden-and-build theory (Fredrickson, 2001), positive emotions broaden an individual’s thought-action repertoire, building enduring personal resources. Internal factors such as cognitive and value development can trigger positive emotions, enhancing students’ hedonic wellbeing by broadening their capacities and resilience. External factors, including clear goals and standards, good teaching, and constructive feedback, create a positive learning environment that reinforces these positive emotions. This helps students associate their academic experience with pleasure and contentment, elevating their overall satisfaction—a key component of hedonic wellbeing.

Social wellbeing involves the quality of relationships and a sense of belonging within the university community. Our study suggests that internal factors, particularly social development including communication skills, collaboration abilities, and intercultural competence, play a crucial role in shaping social wellbeing. Students who possess strong social skills are better equipped to communicate effectively, resolve conflicts, and build trust with teachers and peers, thereby fostering their social wellbeing. Regarding external factors, our study indicates that a supportive learning environment characterized by constructive feedback and clear guidance is essential for fostering a sense of belonging among students. Such an environment enables psychological safety (Edmondson, 1999). When students feel psychologically safe and supported, they are more likely to engage openly, share ideas, and participate actively in the academic community, enhancing their social wellbeing.

Our findings echo the existing literature by emphasizing the important role of student learning and development in fostering eudaimonic, hedonic, and social wellbeing, while adding new value by contextualizing these findings within East Asian culture, which prioritizes collective goals, academic achievement, and social harmony (Choi and Nieminen, 2012; Ho, 2020). The particularly strong prediction of eudaimonic and social wellbeing by internal learning experiences may reflect cultural values in East Asian education systems that stress self-discipline, purposeful striving, and communal connectedness. This cultural orientation may amplify the impact of cognitive, social, and value development on students’ sense of meaning and belonging. Overall, the results underscore the importance of both holistic student development (e.g., cognitive, social, and value dimensions) and a supportive learning environment (e.g., clear guidance and constructive feedback) in promoting wellbeing among Asian students. At the same time, the cross-cultural relevance of these findings suggests that the relationship between learning experiences and wellbeing may reflect a broader pattern that extends across diverse cultural contexts.

### 4.2 Influences of internal and external factors on students’ wellbeing

Extending previous findings that different personal attributes might have differential influences on student development (Yu et al., 2018), our study reveals a diversified influence of students’ learning experiences on wellbeing. Findings from our study indicate that internal factors have a stronger influence on students’ wellbeing compared to external factors. This can be attributed to the inherently intrapersonal nature of internal factors. Cognitive, social, and value developments are deeply rooted in students’ personal growth and self-perception. Cognitive development significantly predicts all three wellbeing dimensions—hedonic, eudaimonic, and social, by fostering critical thinking and self-reflection, which are essential for regulating oneself and enhancing academic performance (Ghanizadeh, 2017; Guamanga et al., 2024). Social development predicts eudaimonic and social wellbeing, enabling students to build meaningful relationships and a sense of belonging and satisfaction (Bye et al., 2019), though it does not significantly predict hedonic wellbeing. One possible explanation is that hedonic wellbeing—focused on pleasure—is more influenced by individual achievement or external rewards than by interpersonal skills, which may contribute more to deeper, long-term wellbeing like purpose or belonging. Value development predicts all three dimensions, and is closely linked to wellbeing by helping students enhance their positive affect across emotional, social, moral, and intellectual domains (Lovat, 2010; Joshanloo and Ghaedi, 2009). These deeply ingrained personal aspects of development are likely to have a more profound and lasting impact on wellbeing than external factors.

External factors, while perceived as less influential, also significantly impact wellbeing. Findings of our study highlight the wellbeing benefits of external factors such as clear goals and standards, good teaching, assessment for understanding, and feedback from teachers. Our findings expand on previous qualitative research (e.g., Upsher et al., 2023; Baik et al., 2019) on the influence of external factors on student wellbeing by providing quantitative evidence. Clear goals and standards are particularly predictive of all three dimensions of wellbeing—hedonic, eudaimonic, and social. They provide students with a sense of direction in their learning and practice, which helps develop self-efficacy (Bandura, 1986), a crucial component of psychological functioning. When students understand what is expected of them, they are more likely to engage actively in various learning activities. This engagement can lead to greater satisfaction and enjoyment, foster a sense of mastery and personal growth, and strengthen social bonds within the learning community, collectively contributing to students’ hedonic, eudaimonic, and social wellbeing.

Good teaching significantly predicts both hedonic and eudaimonic wellbeing, as suggested by our study. Good teaching contributes to a positive learning environment that fosters student engagement and motivation (Ramsden, 2003). When students perceive their teachers as competent, caring, and supportive, they are more likely to experience enjoyment and satisfaction in their learning journey, thus enhancing their hedonic wellbeing. Good teaching also encourages deep learning (Biggs and Tang, 2011), helping students find meaning and purpose in their academic pursuit, which contributes to their sense of eudaimonic wellbeing. However, good teaching alone may not be sufficient to predict social wellbeing, which involves the quality of relationships and a sense of belonging within the educational community. While good teaching can facilitate positive interactions, it is the broader context of student-teacher and peer relationships, along with institutional support, that plays a more critical role in fostering social connections.

Feedback and assessment practices also play crucial roles in shaping different dimensions of student wellbeing. Our study indicates that feedback from teachers significantly predicts both hedonic and social wellbeing, while assessment for understanding primarily predicts eudaimonic wellbeing. Effective feedback is central to student academic performance (Hattie and Timperley, 2007). Constructive and timely feedback helps students recognize their progress and areas for improvement, boosting their confidence and satisfaction. Engaging in feedback exchanges allows students to develop interpersonal skills and a sense of connection, which are essential components of social wellbeing. Assessment for understanding, on the other hand, strengthens self-regulated learning strategies (Clark, 2012; Nicol and Macfarlane-Dick, 2006) and challenges students to engage deeply with the material and apply their knowledge in meaningful ways.

### 4.3 Limitations and future directions

The study has some limitations. First, the reliance on self-reported data may introduce biases such as social desirability bias or recall bias. Future research could benefit from incorporating objective measures of wellbeing and academic performance. Second, the cross-sectional design—where all measures were collected at a single time point—limits causal interpretations. While SEM allows for the testing of theoretical associations among latent constructs, it does not establish causal direction and remains open to alternative explanations, including bidirectional influences and the potential impact of third variables such as self-efficacy or personality traits. Additionally, the findings may not be generalizable to all university students. Efforts should be made to include more culturally diverse and representative samples in future research. Future research directions could include subgroup analyses—such as by gender or academic discipline—and examine the longitudinal associations between internal and external factors of students’ learning experiences and their wellbeing. Additionally, exploring the role of digital and online learning environments in influencing these factors could provide insights relevant to the increasingly digital nature of higher education.

### 4.4 Practical implications

Findings of this study have several practical implications for universities aiming to enhance student wellbeing, offering both explicit and implicit approaches to foster student wellbeing and flourishing. Explicitly, universities can prioritize student wellbeing by strengthening wellbeing support services, offering workshops and courses to build wellbeing literacy, and creating extracurricular opportunities that allow students to apply wellbeing concepts in real-life contexts. Given the strong link found between learning experiences and eudaimonic wellbeing, these initiatives should particularly emphasize self-determined learning, meaning-driven engagement, and holistic growth.

Implicitly, universities can foster students’ cognitive, social, and value development by embedding these factors into university-wide learning outcomes and curricular design. This includes integrating critical thinking, collaboration, ethics, and civic responsibility into coursework and assessment strategies. Informed by the findings that external factors such as clear goals and standards significantly predict student wellbeing, universities could enhance these areas by offering professional development and communities of practice for faculty, with a focus on student-centered pedagogy and inclusive assessment practices. Furthermore, universities should create a supportive and inclusive environment that fosters social connections and a sense of belonging. Initiatives such as mentorship programs, peer support groups, and inclusive social events can help integrate students from diverse backgrounds and enhance social bonds.

## 5 Conclusion

In conclusion, the present study provides compelling empirical evidence that various university learning experiences significantly influence students’ hedonic, eudaimonic, and social wellbeing. By integrating a multidimensional wellbeing framework with both internal and external learning experience predictors, this study extends the literature with quantitative evidence and highlights the wellbeing benefits of student learning experiences. Overall, the study underscores the need for holistic strategies and tailored approaches to nurture student flourishing in higher education.

## Data Availability

The datasets presented in this article are not readily available because pending approval from the authors’ affiliated institution. Requests to access the datasets should be directed to myzhao@hku.hk.

## References

[ref1] Baik C. Larcombe W. Brooker A. (2019). How universities can enhance student mental wellbeing: the student perspective. High. Educ. Res. Dev. 38, 674–687. doi: 10.1080/07294360.2019.1576596

[ref2] Bandura A. (1986). The explanatory and predictive scope of self-efficacy theory. J. Soc. Clin. Psychol. 4, 359–373. doi: 10.1521/jscp.1986.4.3.359

[ref3] Barkham M. Broglia E. Dufour G. Fudge M. Knowles L. Percy A.. (2019). Towards an evidence-base for student wellbeing and mental health: definitions, developmental transitions and data sets. Couns. Psychother. Res. 19, 351–357. doi: 10.1002/capr.12227

[ref4] Baumann M. Amara M.-E. Karavdic S. Limbach-Reich A. (2014). First-year at university: the effect of academic employability skills and physical quality of life on students' well-being. Work 49, 505–515. doi: 10.3233/WOR-131729, PMID: 24004788

[ref5] Bentler P. M. Bonett D. G. (1980). Significance tests and goodness of fit in the analysis of covariance structures. Psychol. Bull. 88, 588–606. doi: 10.1037/0033-2909.88.3.588

[ref6] Biggs J. Tang C. (2011). Teaching for quality learning at university. Maidenhead, UK: Open University Press.

[ref7] Brooker A. Vu C. (2020). How do university experiences contribute to students' psychological wellbeing? Student Success 11, 99–108. doi: 10.5204/ssj.1676

[ref8] Browne M. W. Cudeck R. (1993). “Alternative ways of assessing model fit” in Testing structural equation models. eds. Bollen K. A. Long J. S. (Newbury Park, CA: Sage Publications), 136–162.

[ref9] Bye L. A. Muller F. Oprescu F. (2019). The impact of social capital on student wellbeing and university life satisfaction: a semester-long repeated measures study. High. Educ. Res. Dev. 39, 898–912. doi: 10.1080/07294360.2019.1705253

[ref10] Campbell F. Blank L. Cantrell A. Baxter S. Blackmore C. Dixon J.. (2022). Factors that influence mental health of university and college students in the UK: a systematic review. BMC Public Health 22:1778. doi: 10.1186/s12889-022-13943-x, PMID: 36123714 PMC9484851

[ref11] Choi S. H. J. Nieminen T. A. (2012). Factors influencing the higher education of international students from Confucian East Asia. High. Educ. Res. Dev. 32, 161–173. doi: 10.1080/07294360.2012.673165

[ref12] Cicognani E. Pirini C. Keyes C. Joshanloo M. Rostami R. Nosratabadi M. (2008). Social participation, sense of community and social well being: a study on American, Italian and Iranian university students. Soc. Indic. Res. 89, 97–112. doi: 10.1007/s11205-007-9222-3

[ref13] Clark I. (2012). Formative assessment: assessment is for self-regulated learning. Educ. Psychol. Rev. 24, 205–249. doi: 10.1007/s10648-011-9191-6

[ref14] Collie R. J. Hascher T. (2024). Student well-being: advancing knowledge of the construct and the role of learning and teaching factors. Learn. Instr. 94:102002. doi: 10.1016/j.learninstruc.2024.102002

[ref15] Cox A. M. Brewster L. (2020). Services for student well-being in academic libraries: three challenges. New Rev. Acad. Librariansh. 27, 149–164. doi: 10.1080/13614533.2019.1678493

[ref17] Curren R. Boniwell I. Ryan R. M. Oades L. Brighouse H. Unterhalter E.. (2024). Finding consensus on well-being in education. Theory Res. Educ. 22, 117–157. doi: 10.1177/14778785241259852

[ref18] de la Fuente-Arias J. (2017). Theory of self-vs. externally-regulated learning™: fundamentals, evidence, and applicability. Front. Psychol. 8:1675. doi: 10.3389/fpsyg.2017.01675 29033872 PMC5627139

[ref16] de Ruyter D. (2024). Flourishing as an aim of higher education. Exploring the aspirations and challenges of the educational philosophy of the University of Humanistic Studies (UvH) as an example. J. Philos. Educ, qhae083.

[ref19] Diener E. (1984). Subjective well-being. Psychol. Bull. 95, 542–575. doi: 10.1037/0033-2909.95.3.542, PMID: 6399758

[ref20] Dodd A. L. Priestley M. Tyrrell K. Cygan S. Newell C. Byrom N. C. (2021). University student well-being in the United Kingdom: a scoping review of its conceptualisation and measurement. J. Ment. Health 30, 375–387. doi: 10.1080/09638237.2021.1875419, PMID: 33567937

[ref21] Durlak J. A. Weissberg R. P. Dymnicki A. B. Taylor R. D. Schellinger K. B. (2011). The impact of enhancing students’ social and emotional learning: a meta-analysis of school-based universal interventions. Child Dev. 82, 405–432. doi: 10.1111/j.1467-8624.2010.01564.x, PMID: 21291449

[ref22] Edmondson A. (1999). Psychological safety and learning behavior in work teams. Admin. Sci. Q. 44, 350–383. doi: 10.2307/2666999

[ref23] Ellyatt W. (2022). Education for human flourishing—a new conceptual framework for promoting ecosystemic wellbeing in schools. Challenges 13:58. doi: 10.3390/challe13020058

[ref24] Eloff I. O’Neil S. Kanengoni H. (2022). “Factors contributing to student wellbeing: student perspectives” in L. Schutte, T. Guse, M. P. Wissing, (eds). Embracing well-being in diverse African contexts research perspectives (Cham: Springer International Publishing), 219–246.

[ref25] Entwistle N. J. Ramsden P. (1983). Understanding student learning. London: Croom Helm.

[ref26] Fredrickson B. L. (2001). The role of positive emotions in positive psychology: the broaden-and-build theory of positive emotions. Am. Psychol. 56, 218–226. doi: 10.1037/0003-066X.56.3.218, PMID: 11315248 PMC3122271

[ref27] Fryer L. K. Zeng L. M. Zhao Y. (2021). Assessing university and programme experiences: towards an integrated Asia Pacific approach. Front. Educ. 6:748590. doi: 10.3389/feduc.2021.748590

[ref28] Ghanizadeh A. (2017). The interplay between reflective thinking, critical thinking, self-monitoring, and academic achievement in higher education. High. Educ. 74, 101–114. doi: 10.1007/s10734-016-0031-y

[ref29] Gilmore A. H. McNeilage A. G. Ashton-James C. E. (2025). A scoping review of factors associated with Australian university student wellbeing. Int. J. Wellbeing 15, 1–45. doi: 10.5502/ijw.v15i1.4063

[ref30] Guamanga M. H. Saiz C. Rivas S. F. Almeida L. S. (2024). Analysis of the contribution of critical thinking and psychological well-being to academic performance. Front. Educ. 9:1423441. doi: 10.3389/feduc.2024.1423441

[ref31] Hall D. A. Juliana J. Manickam M. Sunil Singh A. S. T. Wei S. T. S. Vuong P. A.. (2024). Contributions of positive psychology to higher education across Asia: a scoping review and unifying thematic framework. Asia-Pac. Educ. Res. 33, 1–11. doi: 10.1007/s40299-023-00798-y

[ref32] Hattie J. Timperley H. (2007). The power of feedback. Rev. Educ. Res. 77, 81–112. doi: 10.3102/003465430298487

[ref33] Henderson L. W. Knight T. Richardson B. (2013). An exploration of the well-being benefits of hedonic and eudaimonic behaviour. J. Posit. Psychol. 8, 322–336. doi: 10.1080/17439760.2013.803596

[ref34] Ho S. (2020). “Culture and learning: Confucian heritage learners, social-oriented achievement, and innovative pedagogies” in Diversity and inclusion in global higher education. eds. Sanger C. Gleason N. (Singapore: Palgrave Macmillan).

[ref35] Hu L. Bentler P. M. (1999). Cutoff criteria for fit indexes in covariance structure analysis: conventional criteria versus new alternatives. Struct. Equ. Model. 6, 1–55. doi: 10.1080/10705519909540118

[ref36] Huta V. Waterman A. S. (2014). Eudaimonia and its distinction from hedonia: developing a classification and terminology for understanding conceptual and operational definitions. J. Happiness Stud. 15, 1425–1456. doi: 10.1007/s10902-013-9485-0

[ref37] Jones E. Priestley M. Brewster L. Wilbraham S. J. Hughes G. Spanner L. (2020). Student wellbeing and assessment in higher education: the balancing act. Assess. Eval. High. Educ. 46, 438–450. doi: 10.1080/02602938.2020.1782344

[ref38] Joshanloo M. (2016). Revisiting the empirical distinction between hedonic and eudaimonic aspects of well-being using exploratory structural equation modeling. J. Happiness Stud. 17, 2023–2036. doi: 10.1007/s10902-015-9683-z

[ref39] Joshanloo M. Ghaedi G. (2009). Value priorities as predictors of hedonic and eudaimonic aspects of well-being. Pers. Individ. Differ. 47, 294–298. doi: 10.1016/j.paid.2009.03.016

[ref40] Kember D. Webster B. J. Chan W. S. (2020). Refocusing the 3P model to incorporate a learning and teaching environment and graduate attributes. Educ. Psychol. 40, 592–607. doi: 10.1080/01443410.2020.1732304

[ref41] Kern M. L. Arguís-Rey R. Chaves C. White M. A. Zhao M. Y. (2024). Developing guidelines for program design, practice, and research toward a positive and well-being education practice. J. Posit. Psychol. 20, 219–229. doi: 10.1080/17439760.2024.2352743

[ref9001] Keyes C. L. M. (1998). Social Well-Being. Soc. Psychol. Q. 61, 121–140. doi: 10.2307/2787065

[ref42] Kristjánsson K. (2023). “Flourishing as the aim of education: an outline—and ten remaining problems” in M. A. White, F. McCallum, C. Boyle, (eds). New research and possibilities in wellbeing education (Singapore: Springer Nature Singapore), 267–280.

[ref43] Kristjánsson K. Vander Weele T. J. (2024). The proper scope of education for flourishing. J. Philos. Educ.:qhae056. doi: 10.1093/jopedu/qhae056

[ref44] Kuh G. D. (2009). What student affairs professionals need to know about student engagement. J. Coll. Stud. Dev. 50, 683–706. doi: 10.1353/csd.0.0099

[ref45] Kuh G. D. Pace C. R. Vesper N. (1997). The development of process indicators to estimate student gains associated with good practices in undergraduate education. Res. High. Educ. 38, 435–454. doi: 10.1023/a:1024962526492

[ref46] Lance C. E. Butts M. M. Michels L. C. (2006). The sources of four commonly reported cutoff criteria: what did they really say? Organ. Res. Methods 9, 202–220. doi: 10.1177/1094428105284919

[ref47] Lemyre A. Chrisinger B. W. Palmer-Cooper E. Messina J. P. (2024). Mental wellbeing among higher education students in England during the pandemic: a longitudinal study of COVID-19 experiences, social connectedness and greenspace use. Br. Educ. Res. J. 50, 1281–1307. doi: 10.1002/berj.3976

[ref48] Lindsay B. L. Bernier E. Boman J. Boyce M. A. (2023). Understanding the connection between student wellbeing and teaching and learning at a Canadian research university: a qualitative student perspective. Pedagogy Health Promot. 9, 5–16. doi: 10.1177/23733799221089578

[ref49] Liu C. McCabe M. Dawson A. Cyrzon C. Shankar S. Gerges N.. (2021). Identifying predictors of university students’ wellbeing during the COVID-19 pandemic—a data-driven approach. Int. J. Environ. Res. Public Health 18:6730. doi: 10.3390/ijerph18136730, PMID: 34206579 PMC8296899

[ref50] Lizzio A. Wilson K. Simons R. (2002). University students' perceptions of the learning environment and academic outcomes: implications for theory and practice. Stud. High. Educ. 27, 27–52. doi: 10.1080/03075070120099359

[ref51] Lovat T. (2010). “The new values education: a pedagogical imperative for student wellbeing” in International research handbook on values education and student wellbeing. eds. Lovat T. Toomey R. Clement N. (Dordrecht: Springer), 3–12.

[ref52] Lyubomirsky S. King L. Diener E. (2005). The benefits of frequent positive affect: does happiness lead to success? Psychol. Bull. 131, 803–855. doi: 10.1037/0033-2909.131.6.803, PMID: 16351326

[ref53] Makaremi N. Yildirim S. Morgan G. T. Touchie M. F. Jakubiec A. Robinson J. (2024). Impact of classroom environment on student wellbeing in higher education: review and future directions. Build. Environ. 265:111958. doi: 10.1016/j.buildenv.2024.111958

[ref54] Marsh H. W. Huppert F. A. Donald J. N. Horwood M. S. Sahdra B. K. (2020). The well-being profile (WB-pro): creating a theoretically based multidimensional measure of well-being to advance theory, research, policy, and practice. Psychol. Assess. 32, 294–313. doi: 10.1037/pas0000787, PMID: 31829640

[ref56] Muthén L. K. Muthén B. O. (2017). Mplus version 8 user's guide. 8th Edn. Los Angeles, CA: Muthén & Muthén.

[ref57] Nicol D. J. Macfarlane-Dick D. (2006). Formative assessment and self-regulated learning: a model and seven principles of good feedback practice. Stud. High. Educ. 31, 199–218. doi: 10.1080/03075070600572090

[ref59] Oades L. G. Robinson P. Green S. Spence G. B. (2011). Towards a positive university. J. Posit. Psychol. 6, 432–439. doi: 10.1080/17439760.2011.63482

[ref60] OECD (2012). Better skills, better jobs, better lives: a strategic approach to skills policies. Paris: OECD publishing.

[ref61] Osborn T. G. Li S. Saunders R. Fonagy P. (2022). University students’ use of mental health services: a systematic review and meta-analysis. Int. J. Ment. Heal. Syst. 16:57. doi: 10.1186/s13033-022-00569-0, PMID: 36527036 PMC9758037

[ref62] Plakhotnik M. S. Volkova N. V. Jiang C. Yahiaoui D. Pheiffer G. McKay K.. (2021). The perceived impact of COVID-19 on student well-being and the mediating role of the university support: evidence from France, Germany, Russia, and the UK. Front. Psychol. 12:642689. doi: 10.3389/fpsyg.2021.642689, PMID: 34322053 PMC8311121

[ref63] Ramsden P. (1991). A performance indicator of teaching quality in higher education: the course experience questionnaire. Stud. High. Educ. 16, 129–150. doi: 10.1080/03075079112331382944

[ref64] Ramsden P. (2003). Learning to teach in higher education. 2nd Edn. London: Routledge.

[ref65] Riva E. Freeman R. Schrock L. Jelicic V. Ozer C. T. Caleb R. (2020). Student wellbeing in the teaching and learning environment: a study exploring student and staff perspectives. High. Educ. Stud. 10:103. doi: 10.5539/hes.v10n4p103

[ref66] Ryan R. M. Deci E. L. (2000). Self-determination theory and the facilitation of intrinsic motivation, social development, and well-being. Am. Psychol. 55, 68–78. doi: 10.1037/0003-066X.55.1.68, PMID: 11392867

[ref67] Ryan R. M. Deci E. L. (2001). On happiness and human potentials: a review of research on hedonic and eudaimonic well-being. Annu. Rev. Psychol. 52, 141–166. doi: 10.1146/annurev.psych.52.1.141, PMID: 11148302

[ref68] Ryff C. D. (1989). Happiness is everything, or is it? Explorations on the meaning of psychological well-being. J. Pers. Soc. Psychol. 57, 1069–1081. doi: 10.1037/0022-3514.57.6.1069

[ref69] Schmitz B. Schumacher B. Schwarz M. Feldmann F. (2022). Validation of a German and English version of the revised art-of-living inventory. Eur. J. Psychol. Assess. 38, 124–136. doi: 10.1027/1015-5759/a000650

[ref70] Stallman H. M. Ohan J. L. Chiera B. (2018). The role of social support, being present and self-kindness in university student well-being. Br. J. Guid. Couns. 46, 365–374. doi: 10.1080/03069885.2017.1343458

[ref71] Stanton A. Zandvliet D. Dhaliwal R. Black T. (2016). Understanding students’ experiences of well-being in learning environments. High. Educ. Stud. 6, 90–99. doi: 10.5539/hes.v6n3p90

[ref72] Steger M. F. (2009). “Meaning in life” in Oxford handbook of positive psychology. eds. Lopez S. J. Snyder C. R.. 2nd *Edn* (Oxford, England: Oxford University Press), 679–687.

[ref73] Sun H. Richardson J. T. (2012). Perceptions of quality and approaches to studying in higher education: a comparative study of Chinese and British postgraduate students at six British business schools. High. Educ. 63, 299–316. doi: 10.1007/s10734-011-9442-y

[ref74] Tejada-Gallardo C. Blasco-Belled A. Torrelles-Nadal C. Alsinet C. (2020). Effects of school-based multicomponent positive psychology interventions on well-being and distress in adolescents: a systematic review and meta-analysis. J. Youth Adolesc. 49, 1943–1960. doi: 10.1007/s10964-020-01289-9, PMID: 32683592

[ref75] Turnbull A. (2022). Feeling feedback: screencasting assessment feedback for tutor and student well-being. Law Teach. 56, 105–118. doi: 10.1080/03069400.2021.1968168

[ref76] Upsher R. Nobili A. Hughes G. Byrom N. (2022). A systematic review of interventions embedded in curriculum to improve university student wellbeing. Educ. Res. Rev. 37:100464. doi: 10.1016/j.edurev.2022.100464

[ref77] Upsher R. Percy Z. Cappiello L. Byrom N. Hughes G. Oates J.. (2023). Understanding how the university curriculum impacts student wellbeing: a qualitative study. High. Educ. 86, 1213–1232. doi: 10.1007/s10734-022-00969-8, PMID: 36474929 PMC9716146

[ref78] Vander Weele T. J. (2022). The importance, opportunities, and challenges of empirically assessing character for the promotion of flourishing. J. Educ. 202, 170–180. doi: 10.1177/00220574211026905

[ref79] Vander Weele T. J. Hinton C. (2024). Metrics for education for flourishing: a framework. Int. J. Wellbeing. 14, 3197, 1–35. doi: 10.5502/ijw.v14i1.3197

[ref80] Vázquez-Parra J. C. Suárez-Brito P. Alonso-Galicia P. E. Echaniz-Barrondo A. (2023). Critical thinking and student well-being: an approach in university students. Societies 13:232. doi: 10.3390/soc13110232

[ref81] Webster B. J. Chan W. S. Prosser M. T. Watkins D. A. (2009). Undergraduates’ learning experience and learning process: quantitative evidence from the east. High. Educ. 58, 375–386. doi: 10.1007/s10734-009-9200-6

[ref82] Williams G. M. Pendlebury H. Thomas K. Smith A. P. (2017). The student well-being process questionnaire (student WPQ). Psychology 8, 1748–1761. doi: 10.4236/psych.2017.811115

[ref83] Yu L. Shek D. T. Zhu X. (2018). The influence of personal well-being on learning achievement in university students over time: mediating or moderating effects of internal and external university engagement. Front. Psychol. 8:2287. doi: 10.3389/fpsyg.2017.02287, PMID: 29375421 PMC5767243

[ref84] Zeng L. M. Fryer L. K. Zhao Y. (2023). A comparison of three major instruments used for the assessment of university student experience: toward a comprehensive and distributed approach. High. Educ. Q. 77, 27–44. doi: 10.1111/hequ.12363

[ref85] Zhao Y. Huen J. M. Chan Y. W. (2017a). Measuring longitudinal gains in student learning: a comparison of Rasch scoring and summative scoring approaches. Res. High. Educ. 58, 605–616. doi: 10.1007/s11162-016-9441-z

[ref86] Zhao Y. Huen J. M. Y. Prosser M. (2017b). Comparing perceived learning experiences of two concurrent cohorts under curriculum reform in Hong Kong: a multiple-group confirmatory factor analysis approach. Qual. Assur. Educ. 25, 270–286. doi: 10.1108/QAE-11-2016-0070

[ref87] Zou D. Lin Z. Chen C. Yu H. (2024). Factors affecting the wellbeing of mid-achieving university students: a case study from China. Front. Psychol. 15:1465209. doi: 10.3389/fpsyg.2024.1465209, PMID: 39559696 PMC11570998

